# Antidotal activity of *Averrhoa carambola* (Star fruit) on fluoride induced toxicity in rats

**DOI:** 10.2478/intox-2014-0014

**Published:** 2014-11-15

**Authors:** Rupal A. Vasant, A. V. R. L. Narasimhacharya

**Affiliations:** 1 Centre for Interdisciplinary Studies in Science and Technology, Sardar Patel University, Vallabh Vidyanagar, Gujarat, India; 2 Laboratory for Animal Sciences, Department of Biosciences, Sardar Patel University, Vallabh Vidyanagar, Gujarat, India

**Keywords:** fluoride, star fruit, carbohydrate metabolism, lipid metabolism, oxidative stress

## Abstract

Consumption of fluoride leads to several physiological disturbances in carbohydrate, lipid and antioxidant metabolisms. *Averrhoa carambola* L. fruit (Star fruit) is a commonly consumed fruit in tropical countries and is an ingredient in folklore medicines. As the fruits have high polyphenolic and antioxidant contents, the present study was undertaken to investigate the potential of star fruit as a dietary supplement in attenuating the fluoride induced hyperglycemia, hypercholesterolemia and oxidative stress in laboratory rats. A four-week exposure to fluoride caused sustained hyperglycemia, hyperlipidemia and oxidative stress and, when the diet was supplemented with star fruit powder, carbohydrate, lipid and antioxidant profiles were restored significantly. It is surmised that the antihyperglycemic, antihypercholesterolemic and antioxidant activities of star fruit in fluoride exposed rats could be due to the presence of polyphenols, flavonoids, saponins, phytosterols, ascorbic acid and fibers in the fruit, which are all well known regulators of carbohydrate, lipid and antioxidant metabolisms. These findings suggest that star fruit can be used as a dietary supplement in fluoride endemic regions to contain fluoride induced hyperglycemia, hyperlipidemia and oxidative stress.

## Introduction

Fluoride in excess amount can cause several ailments, viz. metabolic disturbances, endocrine dysfunctions and physiological alterations in the body (Jagtap *et al*., 2012; Susheela, 2007). Defluoridation of water is the only available option to reduce the fluoride content from water but the techniques are unaffordable and beyond the reach of underprivileged communities in fluoride endemic areas across the globe. Several natural adsorbents such as red soil, charcoal, brick, fly-ash, serpentine, and alum have been used to reduce the fluoride content in water (Chidambaram *et al*., 2003). Novel defluoridation techniques include the use of leaves of *Azadirachta indica, Ficus religiosa* and *Acacia catechu* and tamarind seeds (Jamode *et al*., 2004; Murugan and Subramanian, 2002, 2006). Apart from these techniques, reports also indicate the utility of plant products, for example tamarind fruit pulp, seeds of *Moringa oleifera,* bark extracts of *Terminalia arjuna* and black berry juice as possible therapeutic agents in amelioration of fluoride toxicity (Hassan and Yousef, 2009; Ranjan *et al*., 2009; Sinha *et al*., 2007). Additionally, plant metabolites such as a 43 kD protein isolated from *Cajanus indicus,* quercetin and curcumin have been shown to ameliorate the fluoride induced oxidative stress and improve the functions of the liver, kidney and erythrocytes (Manna *et al*., 2007; Nabavi *et al*., 2012a, 2012b, c).

*Averrhoa carambola* L., (F: Oxalidaceae; star fruit/carambola) is believed to have originated in Sri Lanka. The plant has been cultivated in Southeast Asia and Malaysia for many centuries (Morton, 1987). In Ayurveda, the ripe fruit is considered digestive and tonic. In India, the ripened fruits are also used to halt hemorrhages and to relieve bleeding hemorrhoids, useful as a treatment for fever, eczema, hemorrhages, hemorrhoids and diarrhea (Morton, 1987). Phytochemical investigation revealed the presence of flavonoids, proanthocyanidins, (–)-epicatechin and vitamin C content in the fruit (Leong and Shui, 2002; Shui and Leong, 2004; Tiwari *et al*., 1979). *In vitro* and *in vivo* studies indicated that star fruit inhibited cytochrome P450 3A activity (Hidaka *et al*., 2004, 2006). Besides, the insoluble fibers isolated from the pomace of star fruit exhibited potent hypoglycemic activities *in vitro*: the insoluble fibers adsorbed glucose efficiently, retarded glucose diffusion and delayed the release of glucose from starch and inhibited the activity of α-amylase (Chau *et al*., 2004a). Further, consumption of water-insoluble fiber-rich fractions of star fruit pomace elevated fecal total lipids, fecal cholesterol and fecal bile acids excretion along with a reduction in serum triacyglycerol, serum and hepatic total cholesterol in hypercholesterolemic hamsters (Chau *et al*., 2004b). The phytochemical analyses and medicinal properties of star fruit are well documented but the role of star fruit in cases of fluoride toxicity has not been studied.

## Materials and methods

### Averrhoa carambola fruit powder preparation and analysis

Fruits of *Averrhoa carambola* were procured from the local market of Vallabh Vidyanagar and authenticated by our faculty taxonomist Dr. A. S. Reddy. The powder of star fruit was prepared by Aum Agri Pvt. Ltd (Baroda, Gujarat) by freeze drying method and stored in an air-tight container. The fiber content of the fruit powder was determined (Thimmaiah, 1999) after petroleum ether extraction followed by acid and alkaline treatment. Saponin and phytosterol contents of the fruit powder were determined using ferric chloride-sulfuric acid and vanillin-sulfuric acid methods, respectively (Ebrahimzadeh & Niknam, 1998; Goad & Akihisha, 1997). The polyphenol and flavonoid contents were measured using Folin-Ciocalteu and Vanillin sulfuric acid reagents (Thimmaiah, 1999). The total ascorbic acid content was estimated using 2, 4-dinitrophenyl hydrazine reagent (Schaffert & Kingsley, 1955). Total antioxidant power in terms of FRAP value was determined using 2, 4, 6- tripyridyl-s-triazine (TPTZ) reagent (Benzie & Strain, 1996).

### Animals and diet

Colony bred male Albino rats (*Charles Foster*; 200–250 g bw) housed individually in a well-ventilated animal unit (26±2 °C, humidity 62%, and 12-h light/dark cycle) were supplied water *ad libitum*. The control animals were fed standard (commercial) diet (Pranav Agro Industries, Vadodara, India) and the experimental groups were provided diets with Ac fruit powder replacing the required amounts of standard (commercial) diet. The research protocol followed the guidelines of Institutional Animal Ethics Committee (MoEF/CPCSEA/Reg. 337) and was approved by the Institutional Committee for animal research.

After a 10-day adaptation period, 30 animals were randomly segregated into 5 groups of 6 animals each as follows: Normal control (NC) - normal animals without any treatment; Fluoride control (FC) - 100 ppm sodium fluoride administered through drinking water; FAc I - fluoride administered animals fed diet with 2.5 g/100 g of *A. carambola* fruit powder; FAc II - fluoride administered animals fed diet with 5 g/100 g of *A. carambola* fruit powder; FAc III - fluoride administered animals fed diet with 10 g/100 g of *A. carambola* fruit powder.

At the end of 30 days, the animals were fasted overnight and sacrificed under mild ether anesthesia. Blood was collected by cardiac puncture and plasma was separated and stored at low temperature. Liver and kidneys were excised and kept frozen until analyzed. Fecal matter was collected for biochemical analyses.

### Analytical procedures

#### Plasma glucose, hepatic glycogen, hepatic hexokinase and G-6-Pase activities

Plasma glucose levels were measured by standard kit (Eve's Inn Diagnostics, Baroda, India). Hepatic glycogen was extracted with 30% KOH, and the yield was estimated by anthrone-sulfuric acid method (Seifter *et al*., 1950). The hepatic hexokinase (EC 2.7.1.1) was determined, based on the reduction of NAD^+^ through a coupled reaction with glucose-6-phosphate dehydrogenase (Brandstrup *et al*., 1957). Glucose-6-phosphatase (EC 3.1.3.9) activity was assayed by measuring the inorganic phosphate liberated from glucose-6-phosphate (Baginsky *et al*., 1974).

#### SGOT, SGPT, ACP, ALP activities and FRAP

Serum glutamate oxaloacetate (SGOT) and pyruvate (SGPT) transaminases, acid and alkaline phosphatases (ACP, ALP) levels were determined using standard kits (Eve's Inn Diagnostics, Baroda, India). The FRAP (ferric reducing ability of plasma) value of the animals was measured by the method of Benzie and Strain (1996).

#### Plasma and hepatic lipid profiles

Plasma total cholesterol (TC), HDL cholesterol (HDL-C) and triglycerides (TG) were measured by standard kits (Eve's Inn Diagnostics, Baroda, India) and the plasma total lipid (TL) content was estimated by sulphophosphovanillin method (Frings *et al*., 1972). Low-density lipoprotein cholesterol (LDL-C), very low-density lipoprotein cholesterol (VLDL-C), and atherogenic index of plasma (AIP) were calculated (Friedewald *et al*., 1972).

The total lipid content (TL) was determined gravimetrically using chloroform/methanol (2:1) liver extracts (Folch *et al*., 1957). The same extracts were also used for estimation of total cholesterol (TC) and triglycerides (TG) using standard kits (Eve's Inn Diagnostics, Baroda, India).

#### Hepatic HMG-CoA reductase and bile acid profile

Hepatic HMG-CoA reductase (EC 1.1.1.34) activity was measured in terms of the ratio of HMG-CoA to mevalonate (Rao & Ramakrishnan, 1975) as HMG-CoA reductase activity is inversely proportional to the ratio of HMG-CoA / mevalonate. Bile acid content was estimated using vanillin-phosphoric acid reagent (Snell & Snell, 1953).

#### Fecal cholesterol and bile acid content

The fecal cholesterol and bile acids were extracted using alkaline-methanol medium and cholesterol was estimated (Kaiek *et al*., 1984). A portion of the extract was acidified and used for bile acid estimation (Snell & Snell, 1953).

#### Hepatic and renal tissue lipid peroxidation and antioxidant profiles

The hepatic and renal lipid peroxidation was determined by the thiobarbituric acid (TBA) assay (Ohkawa *et al*., 1979). The hepatic and renal total ascorbic acid and reduced glutathione contents were estimated using methods of Schaffert and Kingsley (1955) and Jollow *et al*., (1984). Catalase (CAT; EC 1.11.1.6), glutathione peroxidase (GPx; EC 1.11.1.9) and superoxide dismutase (SOD; EC 1.15.1.1) activities were measured in both hepatic and renal tissues following the standard methods (Aebi, 1974; Flohe & Gunzler, 1984; Kakkar *et al*., 1984).

All chemicals used were of analytical grade (SISCO Research Laboratories, Mumbai, India) and all the measurements were taken using Elico ^®^ SL 171 Mini Spec (Hyderabad, India).

### Statistical Evaluation

Data are presented as mean ± SEM (n=6). One-way analysis of variance (ANOVA) with Tukey's significant difference post hoc test was used to compare differences among groups. Data were statistically analyzed using Graph Pad Prism 3.0 statistical software. The *p-*values <0.05 were considered statistically significant.

## Results

The phytochemical analysis of the fruit powder indicated the presence of fiber (3.8 g%), phytosterols (5.06 g%), saponins (3.77 mg%), polyphenols (1.76 g%), flavonoids (0.277 g%) and ascorbic acid (0.088 g%).

**Figure 1:**
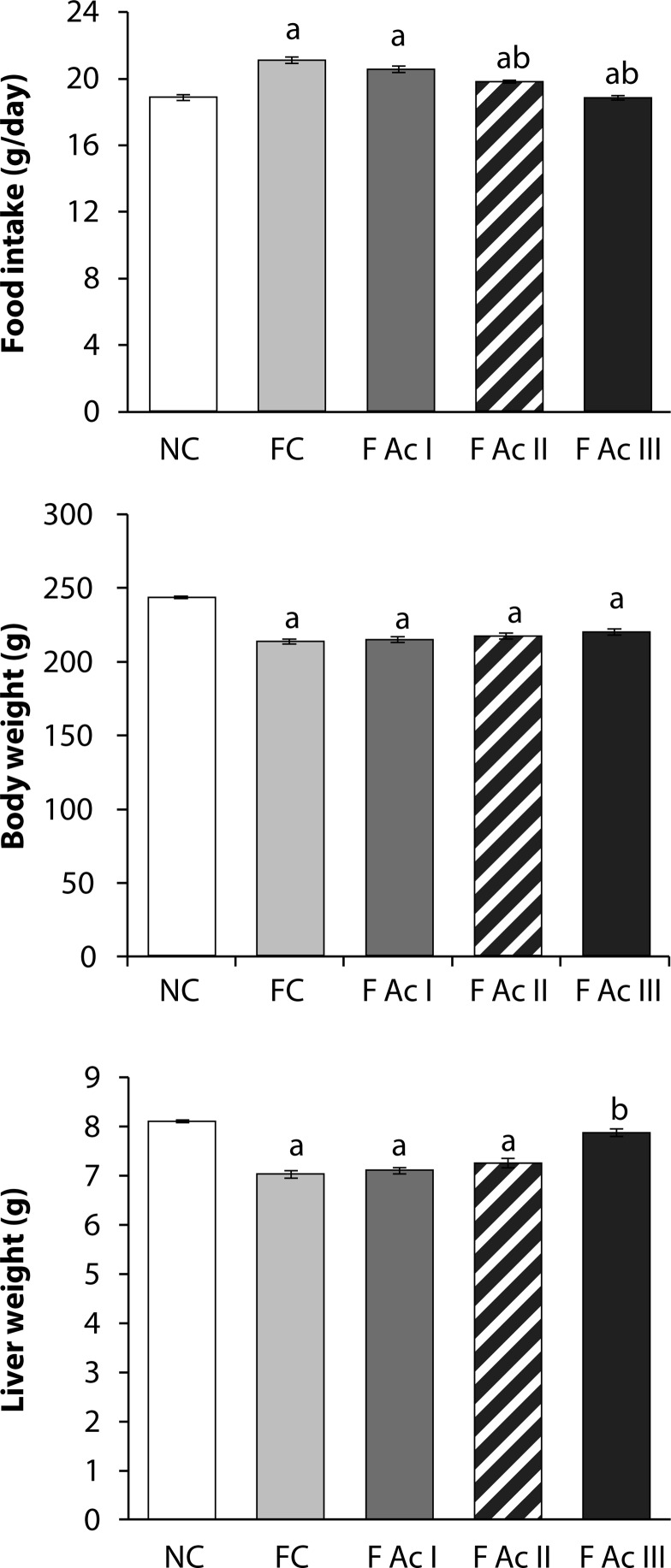
Food intake, body and liver weights. Values are mean ± SEM (n=6); **a -** compared with NC, **b -** compared with FC group

Fluoride exposed animals lost their body and liver weights significantly although the food intake increased. Addition of *A. carambola* fruit powder to the diet of these animals elevated the body and liver weights (3 and 12% respectively) and reduced the food intake (11%) (Figure 1).

**Figure 2:**
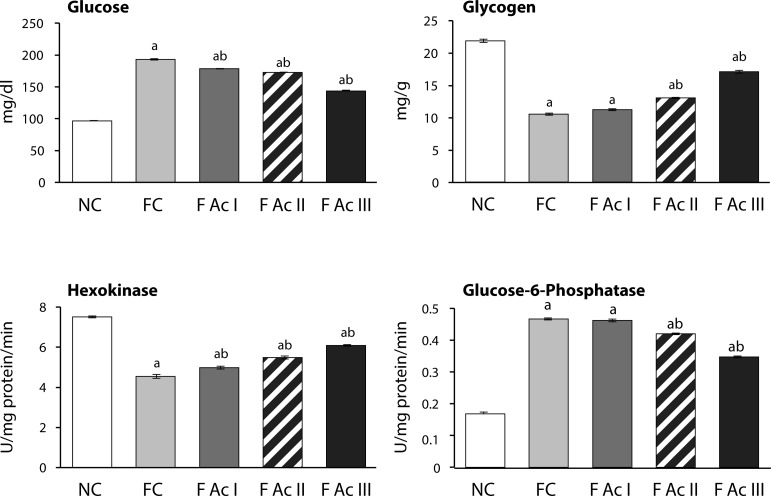
Plasma glucose and hepatic carbohydrate profiles. Values are mean ± SEM (n=6); a - compared with NC, b - compared with FC group

The FC group registered significant elevation in plasma glucose levels (99%), hepatic G-6-Pase activity (175%) and decline in hepatic glycogen content and hepatic hexokinase activity (52% and 40% respectively). In the FAc groups both fasting blood glucose levels and G-6-Pase activity decreased while the hepatic glycogen content and hexokinase activity increased in a dose-dependent manner (Figure 2).

**Figure 3:**
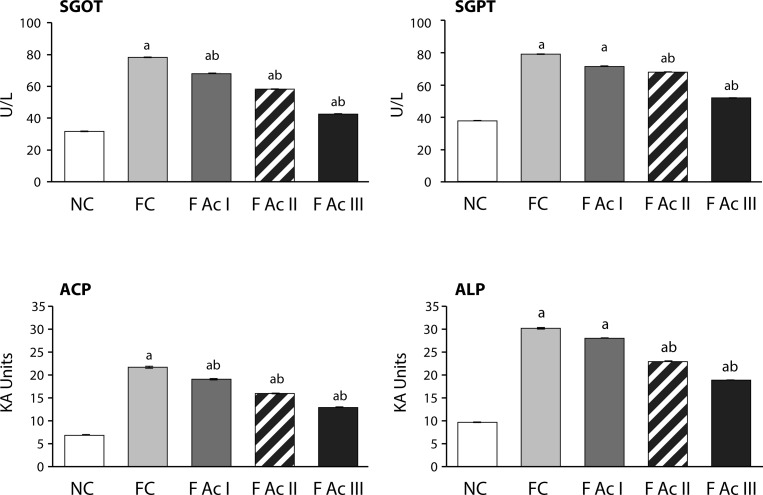
Serum enzymatic profiles. Values are mean ± SEM (n=6); a - compared with NC, b - compared with FC group

While fluoride exposure significantly increased the activities of SGOT, SGPT, ACP, and ALP and decreased the ferric reducing ability of plasma (FRAP) in experimental animals, addition of Ac fruit powder to the diet improved the hepatic functions (Figure 3; Table 1).

**Table 1:** Effect of *A. carambola* plasma total lipids, atherogenic index, Hepatic HMG-CoA and FRAP values.

Parameters	NC	FC	F Ac I	F Ac II	F Ac III
**TL** (mg/dl)	317.37±0.76	470.17±0.63^a^ (+48.14)	445.60±1.08^a^^b^ (-5.22)	390.31±1.81^a^^b^ (-16.98)	347.77±0.81^a^^b^ (-26.03)
**AIP** (mg/dl)	1.58±0.01	3.33±0.03^a^ (+110.76)	3.04±0.02^a^^b^ (-8.71)	2.31±0.02^a^^b^ (-30.63)	1.65±0.01^b^ (-50.45)
HMG-CoA reductase*	3.87±0.02	8.10±0.86^a^ (-109.30)	7.68±0.14^a^ (+5.18)	6.93±0.10^a^ (+14.44)	4.98±0.45^b^ (+38.52)
**FRAP** (µmole/L)	286.12±0.35	159.49±0.33^a^ (-44.26)	195.67±0.38^a^^b^ (+22.68)	219.96±0.20^a^^b^ (+37.91)	246.46±0.38^a^^b^ (+54.53)

Values are represented as mean ± SEM (n=6).

a Indicates the comparison with normal control group

b Denotes the comparison with fluoride control group at *p*<0.05; Percent changes (figures in parenthesis) in fluoride control group were in comparison with normal control and in those treatment groups were in comparison with fluoride control group

* HMG-CoA reductase activity is inversely proportional to the ratio of HMG-CoA/ mevalonate.

**Figure 4:**
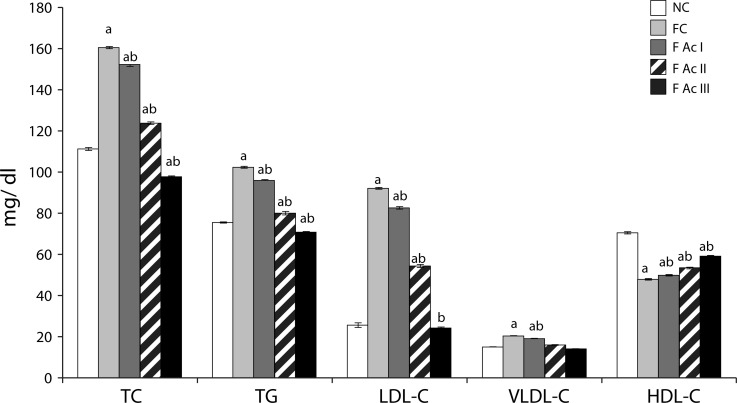
Plasma lipid profiles. Values are mean ± SEM (n=6); a - compared with NC, b - compared with FC group

**Figure 5:**
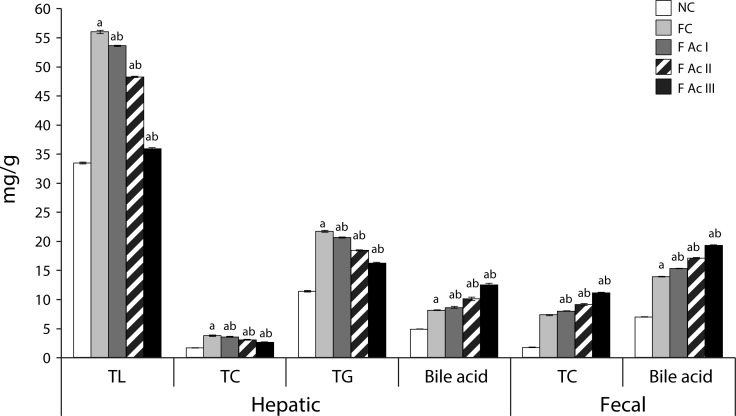
Hepatic and fecal lipid profiles. Values are mean ± SEM (n=6); a - compared with NC, b - compared with FC group

The FC group exhibited significantly high levels of plasma TL, TC, TG, LDL-C, VLDL-C, atherogenic index of plasma (AIP) and, hepatic lipids-TL, TC and TG with reduced HDL-C levels. Ac supplemented groups registered significantly lowered levels of plasma and hepatic lipid profiles and improved HDL-C levels (Table 1; Figures 4 & 5).

Exposure to fluoride significantly suppressed the activity of HMG-CoA reductase, as indicated by increased HMG CoA-mevalonate ratio (NC *vs* FC, FAcI – FAcIII). However, addition of Ac powder to the diet caused increased HMG-CoA activity (5%; 14%; 39%). The bile acid production in hepatic tissue and its excretion in fluoride exposed animals increased significantly compared to that in the NC group and further increases were noted in the FAc groups (Table 1; Figure 5).

**Figure 6:**
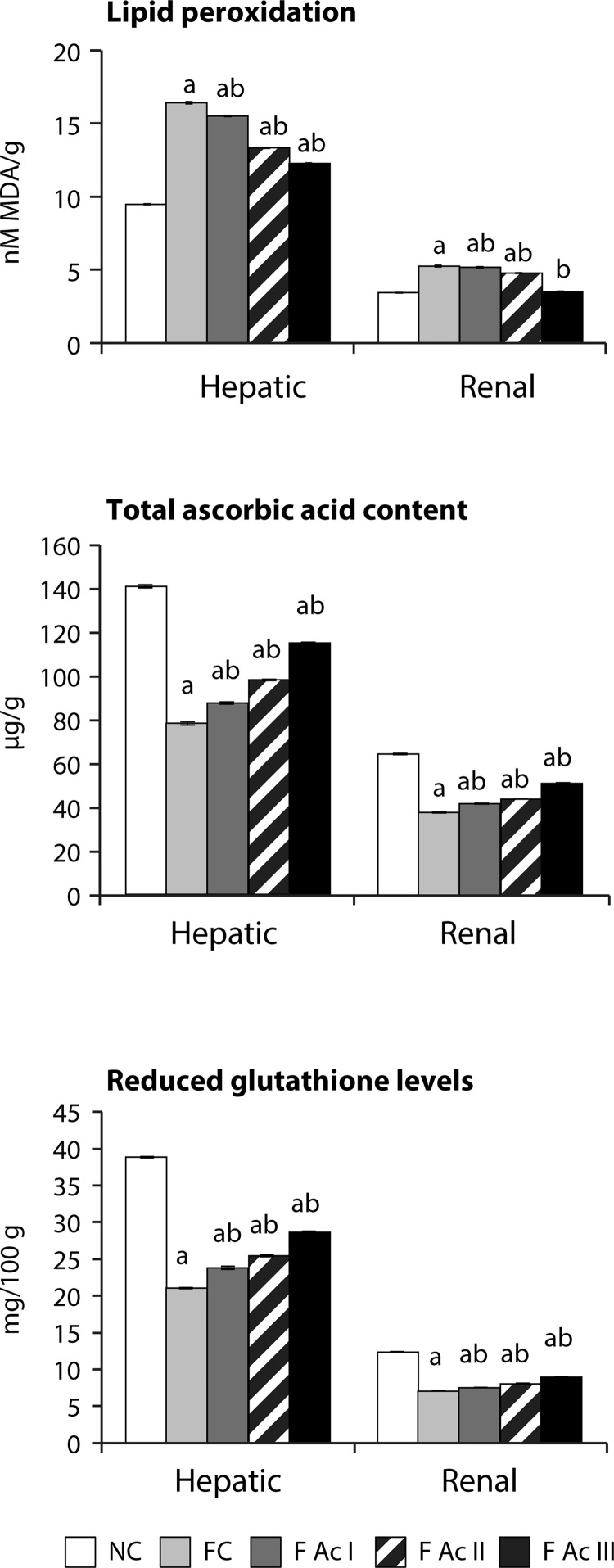
Hepatic and renal lipid peroxidation and non-enzymatic antioxidants. Values are mean ± SEM (n=6); a - compared with NC, b - compared with FC group.

**Figure 7:**
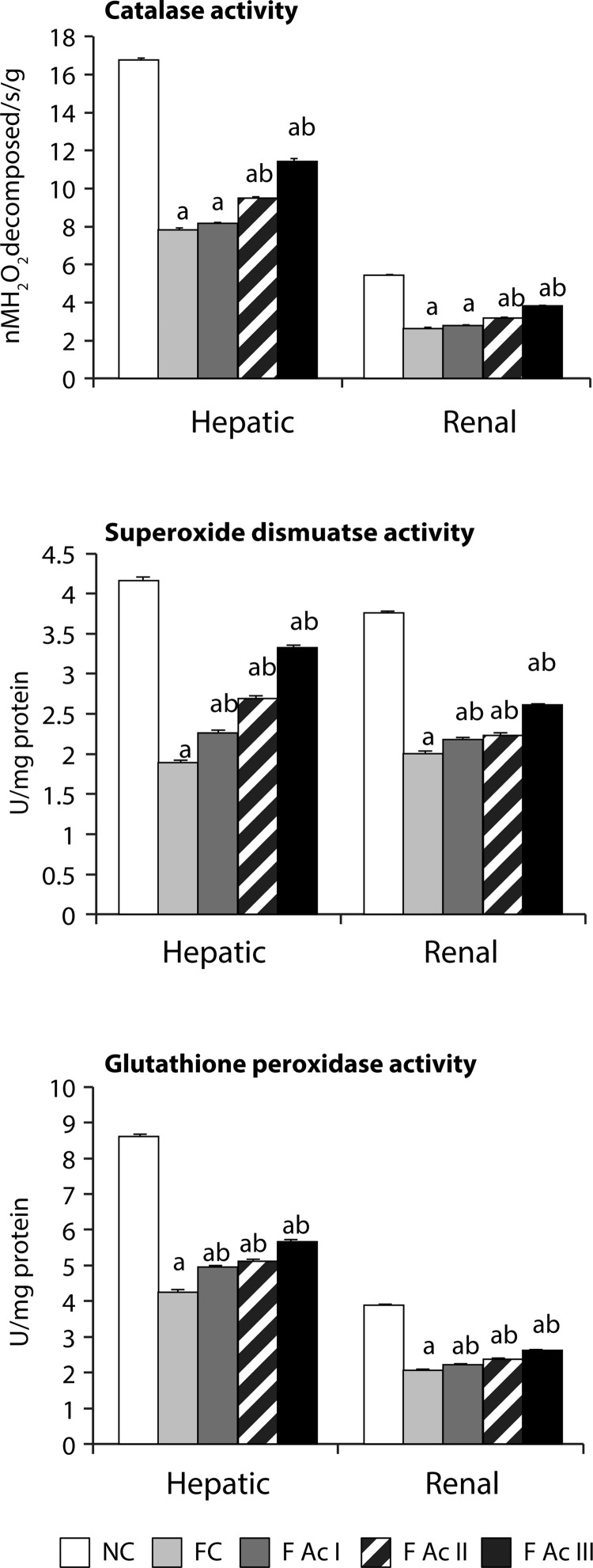
Hepatic and renal enzymatic antioxidants. Values are mean ± SEM (n=6); a - compared with NC, b - compared with FC group.

Both hepatic and renal tissue lipid peroxidation increased (73% and 53% respectively) significantly in fluoride exposed groups. In these groups, the hepatic and renal antioxidant profiles also declined significantly. However, addition of Ac fruit powder to the diet (FAc I – FAc III) improved the antioxidant profiles and decreased the lipid peroxidation in fluoride exposed animals (Figures 6 & 7).

## Discussion

The present investigation reveals for the first time the beneficial effects of *Averrhoa carambola* (Ac) fruit when used as a dietary supplement to regulate fluoride induced alterations in body carbohydrate, lipid and antioxidant metabolism. The positive effects of Ac fruit powder as a food supplement to fluoride exposed rats were found to be dose dependent, *i*.*e*., 10 g/100 g% dose was more potent in ameliorating fluoride induced metabolic alterations when compared to 2.5 and 5 g/100 g% doses.

Fluoride exposed animals lost their body and liver weights significantly although the food intake increased. Addition of *A. carambola* fruit powder to the diet of these animals elevated the body and liver weights and reduced the food intake. The reduction in body and liver weights of fluoride exposed rats could be due to the non-availability of immediate energy resources (carbohydrates) and thus an increase in food intake. These observations are in line with an earlier reported loss of body and liver weights in spite of increased food intake (Yadav *et al*., 2005). Further, the increased food intake in fluoride exposed animals could also be due to a possible suppression of the hunger/satiety center without increasing the energy assimilation, resulting in reduced body and liver weights. With *A. carambola* fruit powder addition, the fluoride induced hunger/satiety-center-inhibition could have been removed and regulated the food intake resulting in increases in body and liver weights.

Exposure to fluoride resulted in significant elevation of plasma glucose levels in FC groups indicating the hyperglycemic activity of fluoride, which is in confirmation with earlier findings (Chlubek *et al*., 2003; Grucka-Mamczar *et al*., 2007; Garcia-Montalvo *et al*., 2009). The increase in hepatic G-6-Pase activity and decline in hepatic glycogen content and hepatic hexokinase activity were countered with A. *carambola* fruit powder addition to the diet. These observations clearly show the antihyperglycemic potential of *A. carambola* to restore the alterations in carbohydrate metabolism caused by fluoride in a highly comparable way as observed in *in-vitro* induced hyperglycemic conditions (Chau *et al*., 2004a). The observed antihyperglycemic activities in fluoride exposed animals administered with *A. carambola* fruit powder supplemented feed could be attributed to the presence of polyphenols, flavonoids, ascorbic acid and saponins in the fruit since these phytochemicals are reported to be antihyperglycemic (Francis *et al*., 2002; Meydani & Hasan, 2010; Oguntibeju, 2008; Yao *et al*., 2004; Zunino *et al*., 2007). Similarly, the altered activities of SGOT, SGPT, ACP, ALP and the declined plasma antioxidant capacity (in terms of FRAP value) in fluoride exposed animals were improved upon the addition of Ac fruit powder to the diet, indicating the restoratory effects of Ac fruit on hepatic tissue.

A long term consumption of fluoride is known to cause hyperlipidemia and hypercholesterolemia (Grucka-Mamczar *et al*., 2004; Strunecka *et al*., 2007) and is also seen in the present study, as especially the FC group exhibited significantly increased lipid profiles both in plasma and hepatic tissue. The substantial decreases in the lipid profiles with an increase in HDL-C and improvement in AI with addition of *A. carambola* fruit powder to the diet clearly indicate the antihyperlipidemic and antiatherogenic properties of *A. carambola* fruit. The increased synthesis of hepatic bile acid and excretion of bile acid and cholesterol (through fecal matter) in fluoride exposed animals supplemented with Ac fruit powder also support the contention that *A. carambola* fruit is antihyperlipidemic and antiatherogenic; an observation similar to the purported effects of fiber-rich fractions of star fruit pomace in hypercholesterolemic hamsters (Chau *et al*., 2004b). The increases in HMG-CoA activity in these animals could be a feedback response to lowered cholesterol levels.

Phytometabolites such as phytosterols, saponins, polyphenols, flavonoids, ascorbic acid and fibers are known to influence the lipid metabolism. For instance, the dietary fibers increase the excretion of cholesterol by interfering with enterohepatic circulation of cholesterol (Arjamandi *et al*., 1992; Moundras *et al*., 1997). Polyphenols, flavonoids and ascorbic acid are reported to be antihyperlipidemic agents as they aid in cholesterol excretion through bile acids (Meydani & Hasan, 2010; Oguntibeju, 2008; Yao *et al*., 2004; Zunino *et al*., 2007). Further, both phytosterols and saponins (present in Ac fruit) could also be responsible for the cholesterol lowering effects of Ac fruit. Phytosterols are involved in inhibition of cholesterol absorption from the intestine due to their greater hydrophobicity and affinity for micelles than cholesterol and displace the intestinal cholesterol (Kritchevsky & Chen, 2005). Saponins precipitate cholesterol from micelles and interfere with enterohepatic circulation of bile acids leading to a reduction in plasma and hepatic cholesterol levels (Francis *et al*., 2002).

The relationship between fluoride intake and oxidative stress is well established (Barbier *et al*., 2010; Strunecka *et al*., 2007). Fluoride generates free radicals like superoxides (O_2_), hydrogen peroxides, peroxynitrites, hydroxyl radicals and other radicals leading to the chemical injury of lipids, proteins and DNA. In the present context, not only the hepatic and renal tissue lipid peroxidation increased significantly but the enzymatic and non-enzymatic antioxidant profiles in liver and renal tissues were found to decrease substantially. *A. carambola* fruit powder inclusion in the diet significantly reduced the lipid peroxidation and enhanced both enzymatic and non-enzymatic antioxidants in a dose-dependent manner. These antiperoxidative effects of *A. carambola* could be due to the inherent antioxidant capacity of the star fruit, *i.e*. FRAP, which is in agreement with the findings of Shui and Leong (2006).

Therefore the results of the present study clearly indicate that the fruits of *A. carambola* are useful as a dietary supplement in regulation of fluoride induced hyperglycemia, hyperlipemia and oxidative stress. Further, this work also suggests that *A. carambola* fruits could be used and promoted as alternative food supplements in fluoride endemic areas.

## ABBREVIATIONS

NC: Normal control
FC: fluoride exposed animals
Ac: *A. carambola*
FAc I: fluoride administered animals fed diet with 2.5% of *A. carambola* fruit powder
FAc II: fluoride administered animals fed diet with 5% of *A. carambola* fruit powder
FAc III: fluoride administered animals fed diet with 10% of *A. carambola* fruit powder
G-6-Pase: glucose- 6-phosphatase
TL: total lipids
TC: total cholesterol
TG: triglycerides
LDL-C: low-density lipoprotein cholesterol
VLDL-C: very low-density lipoprotein cholesterol
HDL-C: high-density lipoprotein cholesterol
AIP: atherogenic index of plasma
SGOT: serum glutamate oxaloacetate transaminase
SGPT: serum pyruvate transaminase
ACP: acid phosphatase
ALP: alkaline phosphatase
FRAP: ferric reducing ability of plasma
TAA: total ascorbic acid
SOD: superoxide dismutase
CAT: catalase
GsH: reduced glutathione
GPx: glutathione peroxidase
